# Back to Basics: Daily Saline Wet Dressings as a Turning Point in Recurrent Pediatric Split-Thickness Skin Graft Failure

**DOI:** 10.7759/cureus.108464

**Published:** 2026-05-07

**Authors:** Jana H Mahdi, Elie Moukawam, Ziad R Hankach, George Ghanime, Ziad Sleiman

**Affiliations:** 1 Plastic and Reconstructive Surgery, Lebanese University Faculty of Medicine, Beirut, LBN; 2 Plastic and Reconstructive Surgery, Lebanese Hospital Geitaoui-University Medical Center, Achrafieh, LBN; 3 Plastic and Reconstructive Surgery, Lebanese University, Beirut, LBN

**Keywords:** graft failure, multidrug-resistant organisms, pediatric trauma, saline dressing, split-thickness skin graft, wound infection

## Abstract

Split-thickness skin grafting (STSG) is a widely used reconstructive technique for managing traumatic soft tissue defects. Successful graft take depends on adequate wound bed preparation and effective infection control. Multidrug-resistant infections remain a major cause of graft failure, particularly in complex pediatric trauma cases.

We report the case of a 12-year-old previously healthy male presenting with bilateral thigh soft tissue defects complicated by multidrug-resistant infections and repeated graft failure. Persistent graft necrosis occurred despite multiple surgical debridements, targeted antibiotic therapy, and negative pressure wound therapy. A modification in postoperative wound care was implemented, including earlier dressing changes and the use of daily saline wet dressings. This approach resulted in successful graft take and complete wound healing, emphasizing the potential role of simple, low-cost wound care strategies in improving outcomes in complex infected wounds.

This case underscores the importance of fundamental wound care principles in the management of complex infected wounds. Early and frequent wound assessment, combined with saline-based dressings, may play a critical role in achieving graft success, even in the setting of multidrug-resistant infections.

## Introduction

Pediatric trauma is a major cause of morbidity worldwide, commonly resulting from high-energy mechanisms such as traffic accidents and falls [1]. These injuries frequently result in extensive soft tissue defects that require complex reconstructive approaches, which remain a significant clinical challenge in pediatric patients [2]. Split-thickness skin grafting (STSG) is a widely used and reliable method for the coverage of traumatic skin defects. However, successful graft take depends heavily on adequate infection control and appropriate postoperative wound care [3].

Bacterial infection is a major contributor to STSG failure. Persistent colonization with multidrug-resistant organisms (MDROs) can impair wound healing by disrupting granulation tissue formation and re-epithelialization, thereby increasing the risk of graft failure despite adequate debridement and targeted antimicrobial therapy [4].

We report a pediatric case of bilateral complex thigh soft tissue loss following traumatic injury, complicated by recurrent multidrug-resistant wound infection and repeated graft failure. This case highlights the challenges of treating contaminated pediatric wounds and the critical role of fundamental wound care practices. It also demonstrates that simple, consistent approaches may still achieve successful graft take despite multiple failures.

## Case presentation

A 12-year-old previously healthy male presented to the emergency department following high-energy blunt trauma after being run over by a truck involving both lower extremities. Initial management was performed at another institution, where he underwent surgical fixation of a right femoral shaft fracture.

**Figure 1:**
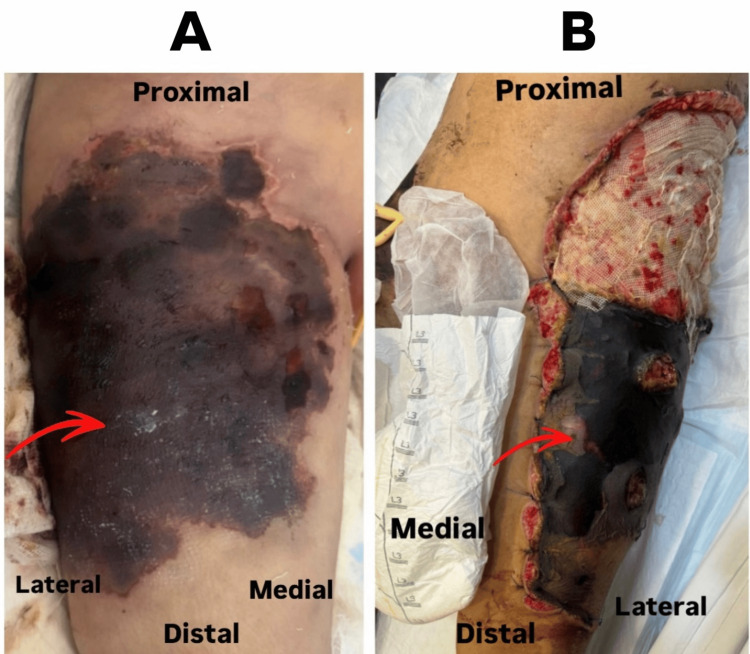
(A) Extensive full-thickness skin necrosis with black eschar involving the right proximal anterolateral thigh (red arrow). (B) Large soft tissue defect of the left proximal anterolateral thigh with exposed muscle and distal necrotic eschar (red arrow).

On presentation to our institution, physical examination revealed extensive open soft tissue injuries involving both thighs. The right proximal anterolateral thigh demonstrated a large wound covered with black necrotic eschar (Figure 1A). The left proximal anterolateral thigh showed a large soft tissue defect with exposed muscle, irregular wound margins, and an inferior necrotic eschar extending toward the knee (Figure 1B). The patient was febrile (39.2°C) but hemodynamically stable.

**Figure 2:**
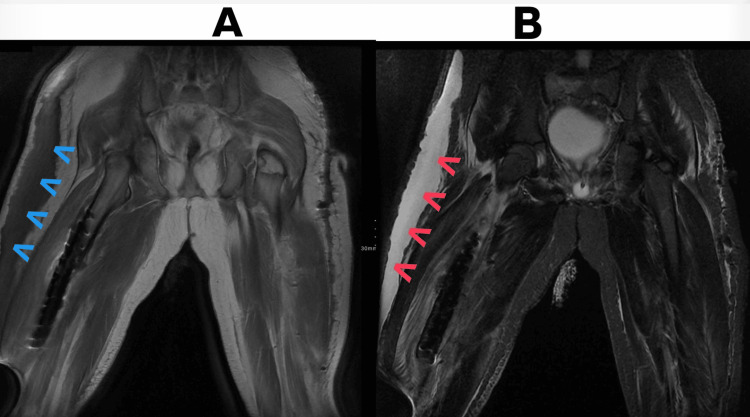
Coronal MRI of the pelvis and thighs demonstrating a Morel-Lavallée lesion. (A) Post-contrast T1-weighted image showing a fluid collection along the lateral right thigh (blue arrows). (B) Proton density fat-saturated image demonstrating corresponding high signal intensity (red arrows).

Magnetic resonance imaging (MRI) of the pelvis and lower extremities demonstrated a right femoral shaft fracture stabilized with a plate and screws, a non-displaced fracture of the right sacral wing, and fractures of the left iliopubic and ischiopubic rami (Figure 2).

**Figure 3:**
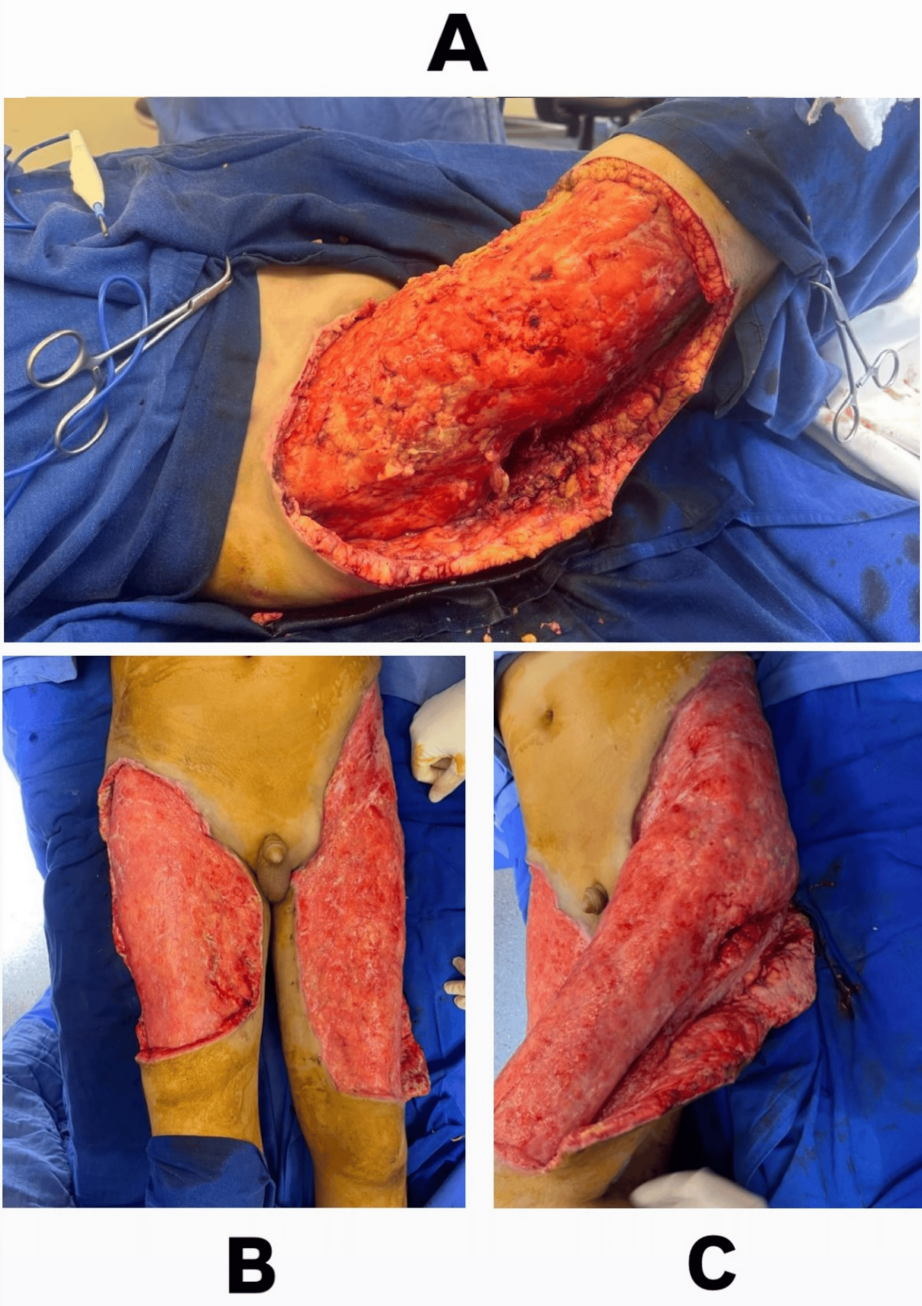
(A) Intraoperative view during initial surgical debridement of the right thigh demonstrating a large soft tissue defect. (B-C) Intraoperative views following repeated debridement showing a clean, viable wound bed with healthy granulation tissue, suitable for split-thickness skin grafting.

The patient underwent surgical debridement of both thighs under general anesthesia on the following day, with excision of all necrotic tissue (Figure 3A). Postoperatively, wound care was initiated using a hyaluronic acid-based dressing (Hyalo4 Start). Wound cultures grew *Acinetobacter baumannii* and *Staphylococcus aureus*, while blood cultures were positive for *S. aureus*. Intravenous piperacillin-tazobactam (4.5 g every eight hours) was initiated according to sensitivity results. Despite appropriate antimicrobial therapy, the wounds remained clinically infected, with persistent foul odor, purulent discharge, and delayed granulation tissue formation.

On hospital day 20, repeat surgical debridement was performed, followed by application of negative pressure wound therapy (NPWT) at 200 mmHg continuous pressure for three days. This higher negative pressure setting was selected due to the heavily contaminated and highly exudative nature of the wound, aiming to promote the formation of a healthy granulation tissue bed in preparation for STSG. However, infection persisted despite multiple debridements, NPWT, and targeted antibiotic therapy. Repeat wound cultures demonstrated MDROs, including *Pseudomonas aeruginosa*, carbapenem-resistant *A. baumannii,* and later *Klebsiella pneumoniae*. This prompted escalation of antibiotic therapy to include vancomycin, imipenem-cilastatin, and subsequently colistin. After a total of three surgical debridements and targeted antimicrobial treatment, the wound beds appeared clinically improved, with healthy granulation tissue and adequate vascularization (Figures 3B-3C).

**Figure 4:**
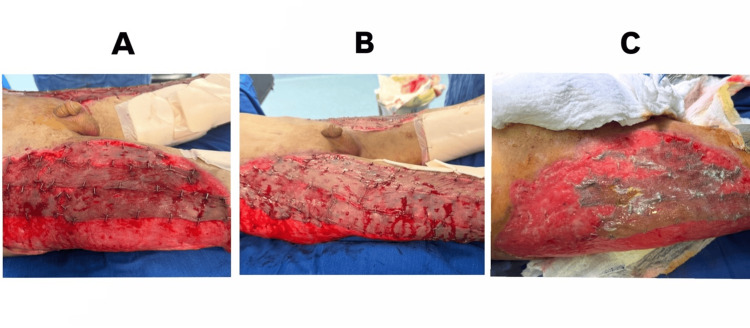
(A-C): Partial graft loss over time following repeated split-thickness skin grafting attempts and graft necrosis with exposed wound bed with surrounding granulation tissue.

On hospital day 40, a 1.5:1 meshed STSG was performed using the ipsilateral anterolateral thigh as the donor site. The first postoperative dressing change was performed on day 5 according to standard protocol. However, the graft demonstrated areas of partial loss with evidence of necrosis and reinfection (Figure 4).

Subsequent grafting procedures were performed at regular intervals based on clinical assessment of wound bed viability and infection control. Instead of a single large graft, multiple smaller STSGs were applied sequentially over separate procedures (a total of six grafting sessions). In addition, postoperative wound care was adjusted, with the first dressing change performed on postoperative day 3, followed by daily wet dressings using sterile normal saline. This approach allowed maintenance of a moist wound environment, closer monitoring, and improved local infection control.

**Figure 5:**
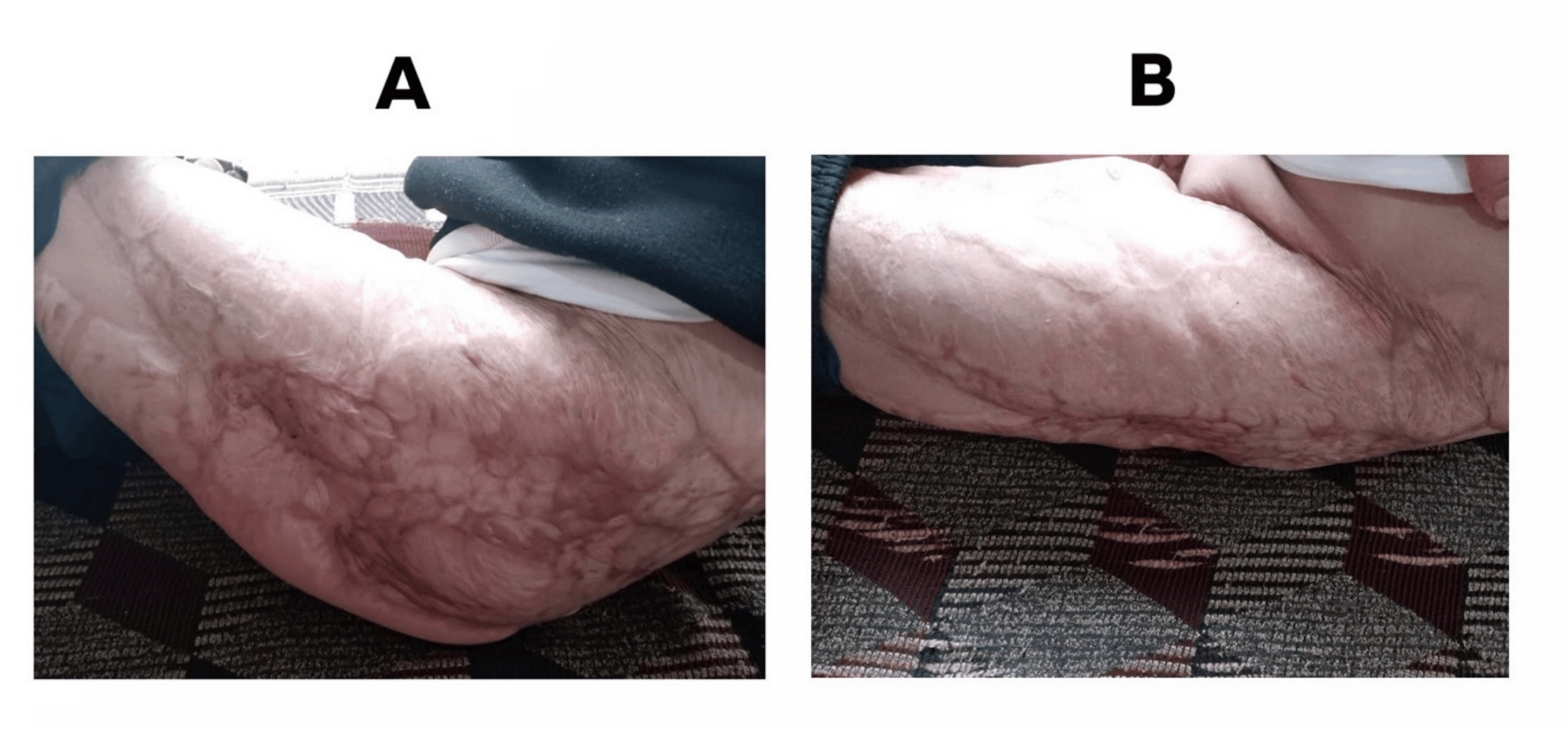
One-year postoperative result following split-thickness skin grafting of the left thigh. (A-B) Complete wound closure and full coverage.

Over a period of approximately three months, progressive wound coverage was achieved using sequential smaller STSGs combined with modified postoperative care. During this period, the wound was initially colonized with MDROs, which were managed with targeted antibiotic therapy. Serial wound care with daily saline dressings, along with oral amoxicillin-clavulanate (1 g twice daily), resulted in effective infection control. Wound cultures obtained prior to the final graft were negative, and complete graft take was achieved without evidence of reinfection (Figures 5A-5B).

## Discussion

STSG is a standard reconstructive technique for coverage of traumatic soft-tissue defects; however, graft success remains highly dependent on adequate wound bed preparation and effective infection control [4]. Bacterial contamination is a well-established factor that impairs wound healing, disrupting the normal phases of tissue repair and increasing the risk of graft failure [5]. In the present case, persistent colonization with MDROs resulted in repeated graft necrosis. Gram-negative bacteria, including *A. baumannii* and *P. aeruginosa*, are known to impair wound healing by delaying granulation tissue formation and interfering with tissue repair mechanisms [6]. This was evident in our case, where repeated wound cultures were positive for these organisms, which are predisposed to delayed wound healing and recurrent graft failure.

NPWT is widely used in the management of complex wounds, especially in pediatric patients, due to its ability to promote granulation tissue formation, reduce edema, and remove exudate [7]. Although NPWT is effective in promoting granulation and reducing edema, its efficacy may be limited in the presence of ongoing MDROs, as observed in this case.

Early assessment and timely surgical intervention are critical in the management of severe pediatric wounds. Current studies recommend early debridement, followed by timely grafting once the wound bed is adequately prepared. Despite the appropriate debridement in this case, persistent infection delayed optimal wound bed preparation, likely contributing to repeated graft failure [7].

Postoperative wound care also plays a crucial role in graft survival. An ideal dressing should maintain a moist environment, reduce bacterial contamination, facilitate autolytic debridement, and protect the wound from further injury [8]. In our case, initial postoperative management with a delayed first inspection on day 5 limited the early detection of graft compromise.

Both the timing and frequency of postoperative dressing changes play a crucial role in graft survival [9]. In this case, the initial postoperative protocol included delayed first inspection until day 5, which may have limited early detection of graft compromise. Following graft failure, the postoperative strategy was modified, with earlier inspection on postoperative day 3 and subsequent daily dressing changes. This adjustment allowed closer monitoring of the wound, earlier detection of infection, and improved local infection control, ultimately contributing to successful graft take.

The choice of dressing is another important factor influencing graft outcomes. Maintaining a moist wound environment has been shown to enhance re-epithelialization and reduce infection risk [9,10]. While various advanced dressings are available, including hydrocolloids, polyurethane films, and hydrofiber dressings, successful graft survival in this case was achieved using simple daily normal saline wet dressings. This emphasizes that basic wound care principles can be as effective as more advanced options when appropriately applied.

In addition, adequate wound irrigation is essential to reduce bacterial load. Evidence suggests that low-pressure saline irrigation is effective while minimizing tissue injury [11]. Both topical and systemic antibiotics play a role in managing wound infections; however, excessive or inappropriate use increases the risk of antimicrobial resistance, which can further complicate healing and contribute to graft failure [12]. This was evident in our case, where MDROs were associated with repeated graft failure.

This case emphasizes that successful management of complex contaminated pediatric wounds relies primarily on strict infection control, timely surgical intervention, and meticulous postoperative monitoring. Even in the era of advanced wound care technologies, adherence to fundamental surgical principles and adaptable, resource-conscious strategies remains essential for achieving reliable graft survival.

## Conclusions

This case highlights the challenges of managing complex pediatric wounds complicated by multidrug-resistant infections and repeated graft failure. Despite the use of advanced wound care modalities, successful graft take was achieved only after modification of postoperative management. Early and frequent dressing changes combined with simple saline-based wound care enabled improved infection control and graft stabilization. These findings emphasize that adherence to fundamental wound care principles can be as important as advanced techniques in achieving successful outcomes in complex infected wounds.
